# Bias Toward Drug-Related Stimuli Is Affected by Loading Working Memory in Abstinent Ex-Methamphetamine Users

**DOI:** 10.3389/fpsyt.2019.00776

**Published:** 2019-10-22

**Authors:** Zoha Deldar, Hamed Ekhtiari, Hamid Reza Pouretemad, Ali Khatibi

**Affiliations:** ^1^Institute for Cognitive Science Studies, Tehran, Iran; ^2^Iranian National Center for Addiction Studies, Tehran University of Medical Sciences, Tehran, Iran; ^3^Institute for Cognitive and Brain Sciences (ICBS), Shahid Beheshti University, Tehran, Iran; ^4^Department of Neurology and Neurosurgery, McGill University, Montreal, QC, Canada; ^5^Centre of Precision Rehabilitation for Spinal Pain (CPR Spine), School of Sport, Exercise and Rehabilitation Sciences, University of Birmingham, Birmingham, AL, United States

**Keywords:** addiction, dual-process models, working memory bias, working memory interference bias, working memory capacity, abstinent ex-methamphetamine users

## Abstract

**Background:** There is a trade-off between drug-related impulsive process and cognitive reflective process among ex-drug abusers. The present study aimed to investigate the impulsive effects of methamphetamine-related stimuli on working memory (WM) performance by manipulating WM load in abstinent ex-methamphetamine users.

**Methods:** Thirty abstinent ex-methamphetamine users and 30 nonaddict matched control participants were recruited in this study. We used a modified Sternberg task in which participants were instructed to memorize three different sets of methamphetamine-related and non–drug-related words (three, five, or seven words) while performing a secondary attention-demanding task as an interference.

**Results:** Repeated-measures ANOVA revealed that reaction times of abstinent ex-methamphetamine users increased during low WM load (three words) compared to the control group (*p* = 0.01). No significant differences were observed during high WM loads (five or seven words) (both *p*’s > 0.1). Besides, reaction times of the experimental group during trials with high interference (three, five, or seven words) were not significantly different compared to the control group (*p* > 0.2).

**Conclusion:** These findings imply that increasing WM load may provide an efficient buffer against attentional capture by salient stimuli (i.e., methamphetamine-related words). This buffer might modify the effect of interference bias. Besides, presenting methamphetamine-related stimuli might facilitate the encoding phase due to bias toward task-relevant stimuli. This finding has an important implication, suggesting that performing concurrent demanding tasks may reduce the power of salient stimuli and thus improve the efficiency of emotional regulation strategies.

## Introduction

Methamphetamine, which is an extremely addictive neurotoxic drug, is the second most used illegal drug after cannabis (1). Prevalence of methamphetamine abuse is 1.2 million people in the United States and 17.2 million people around the world (2). Chronic use of methamphetamine has been associated with multiple physical health problems (e.g., cardiovascular disease), mental health problems (e.g., depression) (3–5), and daily functioning problems (e.g., impulsivity) (6, 7), which can also affect the brain and neurocognitive functions (8–10).

Addiction to methamphetamine—similar to addiction to other substances—is often resistant to conventional interventions (11). Therefore, a critical need exists to address additional and appropriate interventions such as nonpharmacological approaches. In line with this, theoretical models and empirical evidence support a role for the modulation of addiction with cognitive-based approaches (11–17). For example, dual-process models of addiction suggest that addictive behaviors are affected by the dominance of drug-related impulsive processes over the reflective processes (13, 18, 19). Several studies have shown that the drug-related impulsive process is spontaneous, fast, and relatively unconscious, while the reflective process is deliberate, slow, and conscious (13, 18, 19).

There is a trade-off between the drug-related impulsive process and reflective process (11, 13, 15). The drug-related impulsive process is affected by the repeated abuse of drugs (20). Impulsive behaviors in addiction are referred to as behaviors that are associated with selecting an immediate reward, making risky decisions (21), generating memory impairment (22), and showing bias toward salient drug stimuli (12, 23, 24). For example, methamphetamine-related stimuli can involuntarily catch the attention of methamphetamine users (i.e., attentional bias). Attentional bias toward methamphetamine-related stimuli can increase the effect of subjective craving, which may contribute to relapse (25, 26). However, the drug-related impulsive process can be modulated by the reflective system (14, 26). Working memory (WM), which is considered as the main part of the reflective process, can modulate the drug-related impulsive process (14, 26, 27). WM is a temporary storage system that can actively maintain information and manipulate stored information (28). WM is involved in the modulation of the processing of irrelevant information by attentional mechanisms (i.e., the reflective process) (29). However, WM processes can negatively be influenced by emotionally salient stimuli like those related to drugs (18). As a result, the bias toward emotionally salient stimuli can lead to deficits in WM performance (18). Therefore, it is important to understand how WM can modulate the attention given to methamphetamine-related stimuli and vice versa.

Given that WM performance might be impaired in methamphetamine users (10, 30) and in abstinent methamphetamine users, it is plausible that the ability to apply attentional control over methamphetamine-related stimuli is reduced as a result of impaired WM performance. For example, a systematic review on methamphetamine use and cognitive function reported that cognitive domains (e.g., WM performance, attention, cognitive flexibility, inhibitory control, decision making) in methamphetamine users were decreased compared to the control group (31). This reduced cognitive performance was associated with deficits in the brain measures, including lower metabolism, gray matter density, fractional anisotropy, and activation (31). For example, the study of abstinent methamphetamine users showed that WM performance (during a one-back cued response, one- back, two-back, and one-increment tasks) is decreased in abstinent methamphetamine users compared to control group (32). In this study, abstinent methamphetamine users showed increased brain activity in left occipital and right posterior parietal lobe compared to control group, while they showed decreased activity in bilateral putamen/insular cortex and right lateral compared to control group (32). Another study showed a correlation between performance on the delayed recall and increased metabolism in the thalamus in abstinent methamphetamine users compared to the control group (33). Another study also reported a correlation between performance on the word-recall task and hippocampal volume, which was smaller in the abstinent methamphetamine users than in the control group (34). These studies have indicated a decreased cognitive function in methamphetamine users in several domains, including WM performance (31).

Effective cognitive control over addiction encompasses more than simply disengaging attention from methamphetamine stimuli; it is also necessary to maintain attention toward nonmethamphetamine information (14, 35). WM allows us to maintain and prioritize relevant information in the face of irrelevant information (28). Evidence supported the role of WM, and the corresponding processes, in the control of attention (29). To understand effective cognitive control over addiction to methamphetamine, we first need to know the trade-off between the top-down effect of WM in attentional control (reflective process) over methamphetamine-related stimuli and the bottom-up effect of attentional bias in WM (impulsive process) (18).

Studies revealed that automatic attentional mechanisms (i.e., impulsive processes) are not independent of the available processing resources (29, 36). However, investigating the effect of WM capacity (i.e. the ability to actively store information despite ongoing processing, which is an indicator of limited cognitive resources) on the interaction between the reflective process and drug-related impulsive processes is a missing piece in the literature (37–43). Many studies have examined the effect of attentional bias in drug-dependent populations versus control groups (23–24, 25, 44–47). However, according to our knowledge, no study to date has investigated the interactive effect of both bias and WM capacity in abstinent ex-methamphetamine users versus a control group. Therefore, the current study investigated the effect of bias and load on WM maintenance in different ways: first, by showing drug-related words, which are task-relevant stimuli that can facilitate the encoding process; next, by applying an interference task, which can disturb the process of rehearsal and needs to be inhibited; and finally, by increasing WM loads, which can result in greater rehearsal demands. Investigating the effect of these WM manipulations independently in combination with WM load can help determine factors that might contribute to dual-process models of methamphetamine addiction and may lead to the development of effective assessment tools and interventions.

## Methods

### Ethics Approval

All experimental procedures corresponded to the standards set by the latest revision of the Declaration of Helsinki and were approved by the ethical committee of the Institute for Cognitive Sciences Studies, Tehran, Iran. All participants provided written informed consent, acknowledging their right to withdraw from the experiment without prejudice.

Showing methamphetamine cues to participants may increase the possibility of relapse. Concerning this important ethical issue, we used methamphetamine-related words instead of real substances. In addition, participants were monitored in the following weeks for any signs of drug craving, and they also had access to psychological interventions to manage potential drug cravings.

### Participants

Thirty abstinent ex-methamphetamine users (all men, 20–47 years old, experimental group) and 30 participants without a history of addiction or drug abuse (all men, 20–50 years old, control group) were recruited in the current study (**Table 1**). The experimental group was recruited from former methamphetamine-dependent users who were admitted to Vardij Abstinence-Based Residential Centre, Karaj, Iran. This treatment center specializes in amphetamine-type stimulant dependence and is located in a rural area near Tehran—a part of the therapeutic network belonging to Rebirth Society Organization (a nonprofit charity). The abstinent ex-methamphetamine users in this center were relatively homogeneous, and only men were admitted. Participants in the control group (all males) were recruited from employees of Shahid Beheshti University, Tehran, Iran. They reported no history of drug abuse. Both groups were right-handed and were matched for age (20–50 years) and educational level (<12 years of school. Inclusion criteria for the experimental group included having a history of methamphetamine abuse in the past 12 months prior to entering the treatment center (methamphetamine dependence based on *Diagnostic and Statistical Manual of Mental Disorders, Fifth Edition* criteria). The most common mean of drug administration was smoking. Subjects had to be abstinent from any drugs except cigarettes for at least a week before the experiment, with confirmation by urine testing.

**Table 1:** Demographic and substance abuse characteristics.

Descriptive	Experimental group	Control group
Gender (men)	30	30
Age (years)	31.5 ± 1.22	28.07± 1.42
Education (years)	11.97 ± 0.47	12.78 ± 0.5
Duration of meth abstinence (day)	17.26 ± 1.43	—
Duration of meth dependence (months)	45.2 ± 4.87	—

Values are reported as mean ± SEM.

Exclusion criteria for both the experimental group and the control group included any current or past major clinical neurological disorders, central nervous system–effective medication intake, or any major clinical psychiatric disorders (in Axis I, except substance-related disorders). We excluded data of two participants from the experimental group and data of two participants from the control group because of their inaccurate responses to the cognitive task.

### The Modified Sternberg Task With Interference

**Figure 1:**
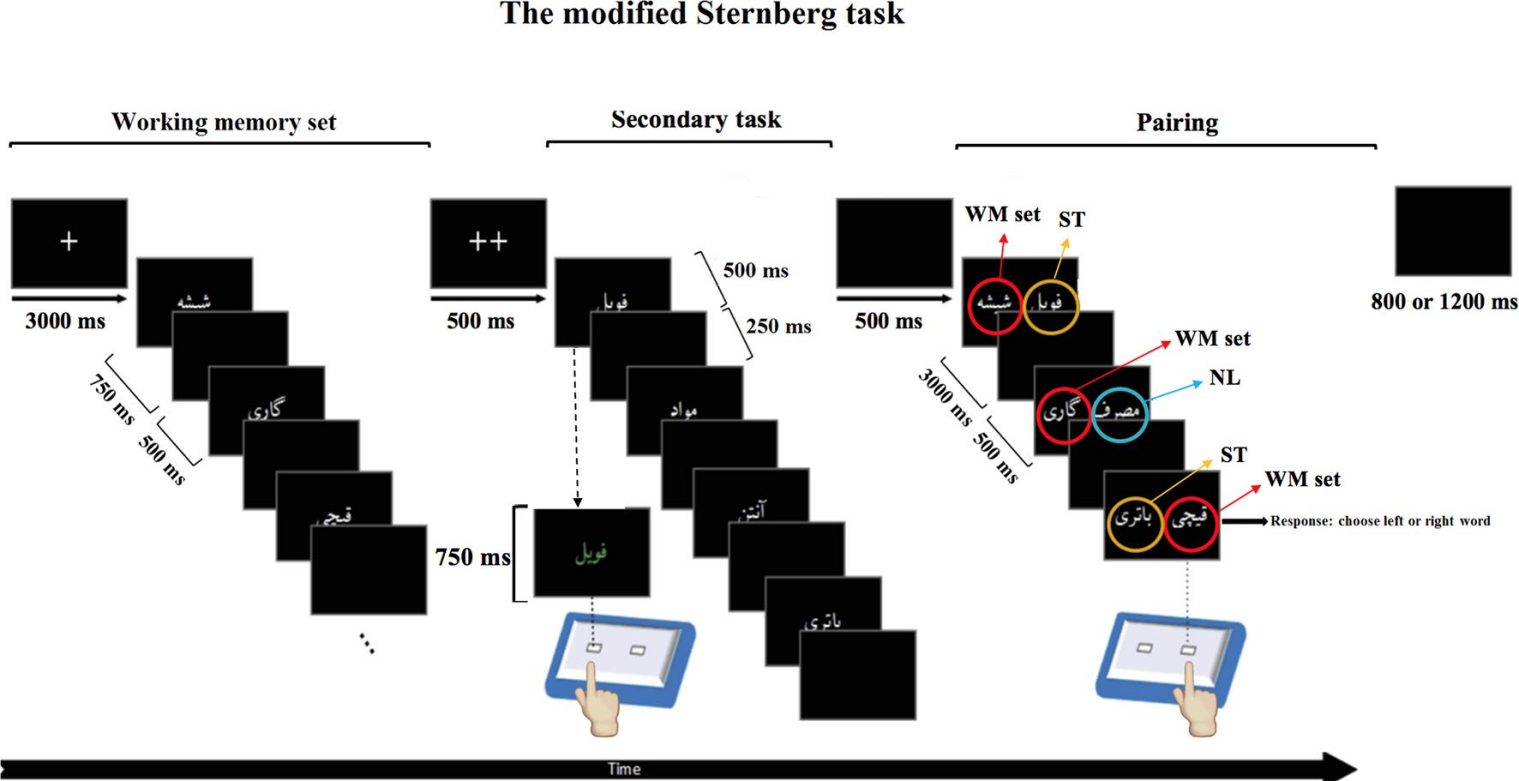
Modified Sternberg task with interference.This Figure is an example of one trial with different responses at different steps of the modified Sternberg task with interference. The modified Sternberg task with interference consisted of three steps. The first step shows a working memory set with three Persian words, out of which one is a methamphetamine-related word (e.g., methamphetamine) and two are non–drug-related words (e.g., cart and scissors). This step is considered as an encoding step. The second step illustrates the secondary task in which the color of the words can change to green or blue (interference step). Subjects have to respond to the color of the word by pressing the corresponding button on the response box (e.g., left for green, right for blue). During the last step, called the pairing step, the participants have to choose the correct word from the working memory set by using the response box (according to their position on the screen; recall step). Numbers represent the display time of the words on the screen (Figure is modified from (48).

To test WM performance of participants, we adopted a modified Sternberg task with interference. The task was designed with MATLAB (The MathWorks, Inc., Natick, MA, United States) and used the Psychtoolbox ran on a Microsoft Windows 7 operating system. The modified Sternberg task fits in the category of a complex span task. It consisted of three steps: memorizing a list of words (encoding step), performing a secondary task (as an interference step), and selecting the memorized word among presented words (retrieval step). In order to obtain different levels of WM load, the Sternberg task included a list of either three, five, or seven words (**Figure 1**) (19).

We selected a list of words, that were validated in a previous study based on their mean of craving and emotional valence (49). This list consisted of 24 Persian words: 12 were selected randomly from a list of methamphetamine-related words (i.e., experimental; ex: methamphetamine, drugs), and 12 were selected randomly from a list of non–drug-related words (i.e., neutral; ex: scissors, carriage). All words had two syllables with a maximum of four letters. They were presented with the same font in white color on a black background screen.

#### Proceeding of the Modified Sternberg Task With Interference

The first step (i.e., WM set) consisted of the presentation of a list of three, five, or seven words (encoding step). Participants had to memorize the presented word list. Words were presented randomly according to methamphetamine-related or non–drug-related content. For example, in the 3-word memory set, there were either two methamphetamine-related words and one non–drug-related word or vice versa. In the 5-word WM set, there were either three methamphetamine-related words and two non–drug-related words or three non–drug-related stimuli and two methamphetamine-related words. Each word was presented for 750 ms with an interstimulus interval of 500 ms. Two fixation crosses (++) were presented in the center of the screen to signal the end of this step (**Figure 1**, left panel).

The second step consisted of a secondary task (as an interference step). In this step, four words were presented one after the other. Two of these four words were methamphetamine-related words, and two were non–drug-related. These words were new and different from the words used in the memory set step. Each word was presented for 500 ms, after which time the font color was changed randomly to either blue or green (750 ms). Participants were asked to indicate which font color was used for the words by pressing the corresponding button on the response box (left button for green, right for blue). After the participant’s response, a black slide was presented for 250 ms, and the next word appeared. One-third of the trials were null; instead of word stimuli, an empty black screen was presented. The total duration of the secondary task was 6 seconds (**Figure 1**, middle panel).

In the third step (i.e., pairing step), each word from the first step was presented once along with a word from the secondary task (ST trial) or a novel list (NL) of methamphetamine-related and non–drug-related words, which had not been presented in that trial, for 3,000 ms. The participant’s task was to choose the word that was presented in the memory set by pressing the corresponding key on the response box (i.e., right button for the word on the right side of the screen, and left button for the word on the left) as fast and as accurate as possible. After the presentation of each pair and response by the participant, the screen was replaced by a black slide for 500 ms, and the next pair appeared on the screen. After all words from the memory set were presented, a single fixation cross was presented in the center of the screen, and the next trial was started. The intertrial time interval was set to be between 800 and 1,200 ms (**Figure 1**, right panel). This pairing step is referred to as retrieval step. A black screen was presented for 500 ms after each probe. At the end of the three probes, a black screen was presented randomly for 800 or 1,200 ms (**Figure 1**, right panel).

Second words (i.e., incorrect words) during the pairing step were selected randomly considering the following restrictions: at least one methamphetamine-related word and one non–drug-related word were required to be among the words. Second words in each probe had a 50% chance of being randomly selected from the ST step of its respective trial (i.e., high interference trials). The remaining second words were again randomly selected among methamphetamine-related and non–drug-related words (NL) not previously presented in its respective trial (i.e., low interference trials).

Regarding the mentioned rules for the presentation of both words, the display in the pairing step included the situations as below:

- methamphetamine-related words (WM set) + methamphetamine-related words (ST);
- methamphetamine-related words (WM set) + methamphetamine-related words (NL);
- methamphetamine-related words (WM set) + non–drug-related words (ST);
- methamphetamine-related words (WM set) + non–drug-related words (NL);
- non–drug-related words (WM set) + methamphetamine-related words (ST);
- non–drug-related words (WM set) + methamphetamine-related words (NL);
- non–drug-related words (WM set) + non–drug-related words (ST);
- non–drug-related words (WM set) + non–drug-related words (NL).

### Experimental Procedure

All subjects participated in one session. The experimental procedure was explained clearly to them at the beginning of the session. Basic demographic information, drug abuse, treatment history, and high-risk behaviors of each subject were recorded during a structured interview by an expert drug counselor. After signing the consent form, participants sat in front of a 13-inch laptop screen at a 60-cm viewing distance in a room with dimmed light to increase their focus on the screen.

The experimental procedure had two different phases: a training phase and a test phase. The goal of the training phase was to learn how to perform the Sternberg task. The training task was designed similarly to the main one, but with different words compared to the main experiment (all of them non–drug-related). After it was sure that participants knew how to perform the task, they proceeded to the testing phase.

Overall, participants performed three conditions, including a condition of three WM words consisting of 72 trials, a condition of five WM words also consisting of 72 trials, and a condition of seven WM words consisting of 72 trials (**Figure 1**). All 24 words appeared equally in the probe; they were also paired with second words (incorrect words) in all types of pairings (i.e., methamphetamine-related words, non–drug-related words, and non–drug-related words from the NL). Each of the 24 words was repeated 27 times during 72 trials. The sequence of words was counterbalanced between participants.

### Statistical Analysis

Data analysis was conducted using SPSS 21 and Statistica v13 (Dell Inc., Tulsa, OK, USA). All results are expressed as the mean ± standard error of the mean, and the statistical threshold was set to *p* ≤ 0.05. A priori hypotheses were tested with *post hoc* analysis (Tukey test) and planned contrasts. The data from trials with null stimuli were excluded from all statistical tests. To analyze the reaction time (RT), trials with incorrect responses were excluded from relevant statistical tests.

#### Bias Caused by Difference Sources

Potential bias, caused by the methamphetamine-related words on the performance of experimental participants, was from different sources and should be separated in the current task paradigm.

- The first bias we considered was the summation of WM interference bias and WM bias during different WM loads (three, five, or seven words). This score was defined as 1/2 * (RT (E − G) + RT (F − H) + RT (G − C) + RT (H − D)). Repeated-measures ANOVA was used to calculate this first bias: the three first bias scores during the different WM loads (three, five, or seven words) were considered as a within-subject factor, and subject group (experimental, control) was considered as the between-subject factor.
- The second bias we referred to was the WM interference bias. This score was defined as (RT(E) + RT(F))/2 − (RT(G) + RT(H)/2. Repeated-measures ANOVA tests were used to obtain this second score; three second bias scores during the different WM loads (three, five, or seven words) were considered as a within-subject factor, and subject group (experimental, control) was considered as the between-subject factor.
- The third bias we referred to was the WM bias. This score was defined as (RT(G) + RT(H)/2 − (RT(C) + RT(D))/2. Repeated-measures ANOVA tests were used in order to calculate this score: three third bias scores during the different WM loads (three, five, or seven words) were considered as a within-subject factor, and subject group (experimental, control) was considered as the between-subject factor.

#### The Effect of Different WM Loads on the Performance of Participants During High Interference Trials

The performance of participants during high interference trials (i.e. the high interference effect caused by the words from the secondary task) was measured during different WM loads (three, five, or seven words). High interference trials included trials from A, C, E, and G conditions. To test the effect of presenting different WM loads (three, five, or seven words) on the performance of participants, two separate repeated-measures ANOVA tests were performed on RTs and accuracy of participants during high interference trials. In this analysis, RTs and accuracy during different WM loads (three, five, or seven words) were considered as a within-subject factor, and subject group (experimental, control) was considered as the between-subject factor.

## Results

### Bias Caused by Difference Sources

#### The First Bias

**Figure 2:**
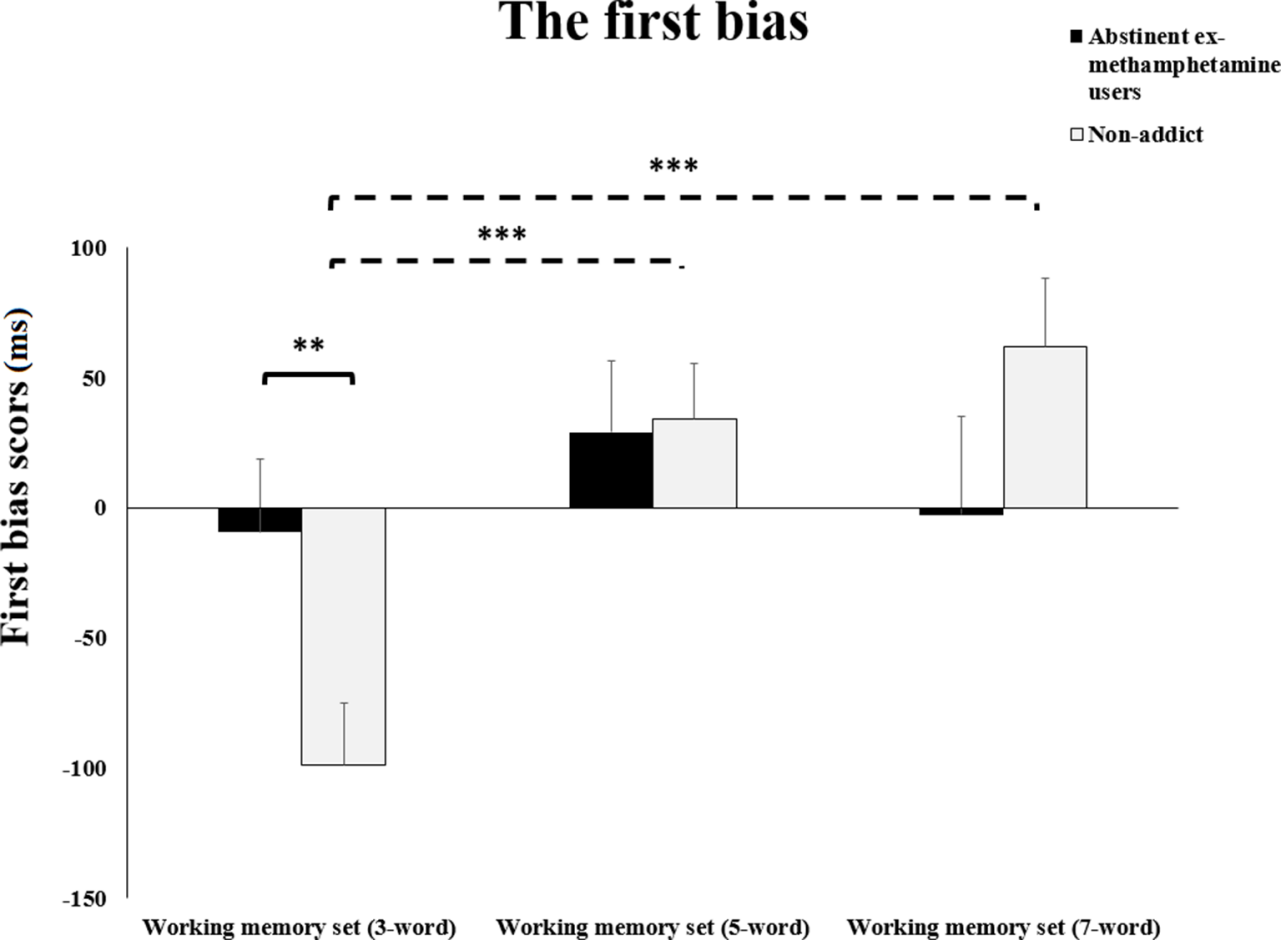
The first bias.The summation of reaction times of working memory interference bias and working memory bias during different working memory loads (three, five, or seven words). Error bars indicate standard error of the mean **p ≤ 0.01; ***p ≤ 0.01.

Repeated-measures ANOVA test showed a significant interaction effect between WM load and subject group on the first bias of RTs (*F*(2, 116) = 3.76, *p* = 0.02). Planned contrasts analysis revealed that mean scores for the first bias RTs of the methamphetamine user group significantly increased during the performance of the 3-word WM compared to the control group (*p* = 0.01). However, no significant difference was observed in mean RTs during the 5- and 7-word WM sets (both *p*’s > 0.1) (**Figure 2**).

Planned comparisons based on our priory hypothesis revealed that mean scores for the first bias RTs of the control group were significantly increased during the performance of the 3-word WM set compared to the 5- and 7-word memory sets (both *p*’s < 0.001). However, no significant difference was observed in mean scores for the first bias RTs of the control group when comparing the 5-word WM sets to the 7-word sets (*p* > 0.2). Additionally, no significant difference was observed in mean scores for the first bias RTs of the experimental group during the 3-word WM set compared to the 5- and 7-word memory sets (*p*’s > 0.2) (**Figure 2**).

#### The Second Bias

Repeated-measures ANOVA test showed no significant interaction effect between WM load and subject group on the second bias of RTs of the experimental group compared to the control group (*F*(2, 116) = 1.97, *p* = 0.14). Planned comparisons based on our priory hypothesis revealed that mean scores for the second bias RTs of the control group were not significantly changed during the performance of the 3-word WM set compared to the 5- and 7-word memory sets (both *p*’s > 0.2). No significant difference was observed in mean scores for the second bias RTs of the control group when comparing the 5-word WM sets to the 7-word sets (*p* > 0.2). Additionally, mean scores for the second bias RTs of the experimental group were not significantly changed during the performance of the 3-word WM set compared to the 5- and 7-word memory sets (both *p*’s > 0.1). No significant difference was observed in mean scores for the second bias RTs of the experimental group when comparing the 5-word WM sets to the 7-word sets (*p* > 0.05).

#### The Third Bias

Repeated-measures ANOVA test showed no significant interaction effect between WM load and subject group on the third bias RTs of the experimental group compared to the control group (*F*(2, 116) = 0.81, *p* = 0.44). Planned contrasts analysis revealed that mean scores for the third bias RTs of the control group were not significantly changed during the performance of the 3-word WM set compared to the 5- and 7-word memory sets (both *p*’s > 0.1). No significant difference was observed in mean scores for the third bias RTs of the control group when comparing the 5-word WM sets to the 7-word sets (*p* > 0.2). Additionally, mean scores for the third bias RTs of the experimental group were not significantly changed during the performance of the 3-word WM set compared to the 5- and 7-word memory sets (both *p*’s > 0.2). No significant difference was observed in mean scores for the second bias RTs of the experimental group when comparing the 5-word WM sets to the 7-word sets (*p* > 0.05).

### The Effect of Different WM Loads on the Performance of Participants During High Interference Trials

#### Reaction Times During Trials With High Interference

**Figure 3:**
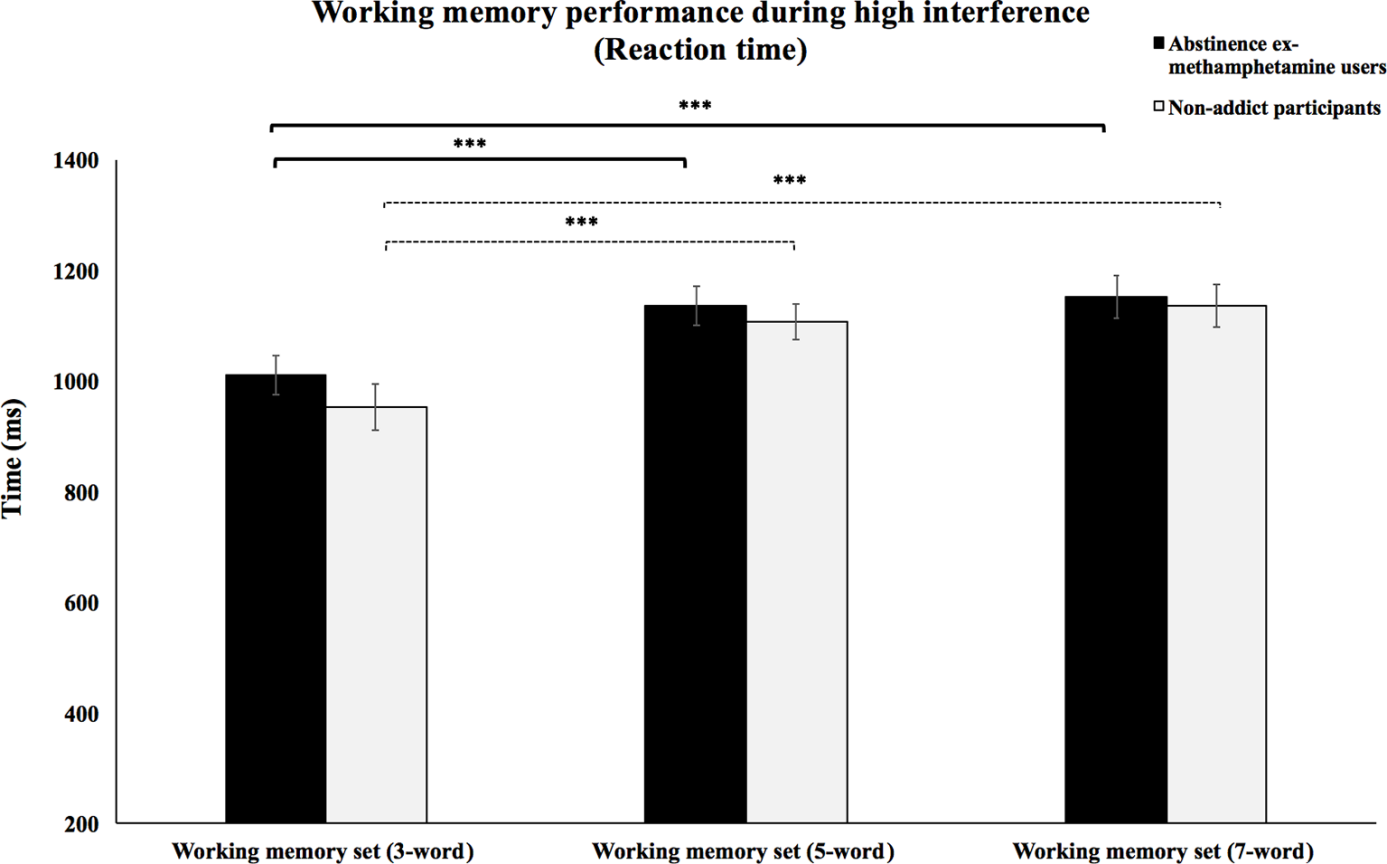
Working memory performance during high interference (reaction times).Mean reaction times of abstinent ex-methamphetamine users and nonaddict control group for the recognition of words from the 3-word working memory sets were compared to the 5- and 7-word working memory sets during trials with high interference. Error bars indicate standard error of the mean ***p ≤ 0.001.

Repeated-measures ANOVA test showed no significant interaction effects between WM load and subject group on the RT of high interference condition (*F*(2, 116) = 0.47, *p* = 0.62). However, priori hypotheses were tested with planned contrasts, and the type I error rate was controlled for using the Bonferroni correction for multiple comparisons. Planned contrasts analysis revealed that the mean RTs of the methamphetamine user group significantly decreased during the performance of the 3-word WM set compared to performing the 5- and 7-word WM sets (*p*’s < 0.001). However, no significant difference was observed in mean RTs during the 5-word WM set compared to mean RTs when performing the 7-word WM sets (*p* > 0.2). The same results were also found in the control group. Mean RTs of the control group during performance of the 3-word WM set compared to the 5- and 7-word memory sets were significantly decreased (*p*’s < 0.001), but no significant difference was observed in mean RTs when comparing the 5-word WM sets to the 7-word sets (*p* > 0.2) (**Figure 3**).

#### Accuracy During Trials With High Interference

**Figure 4:**
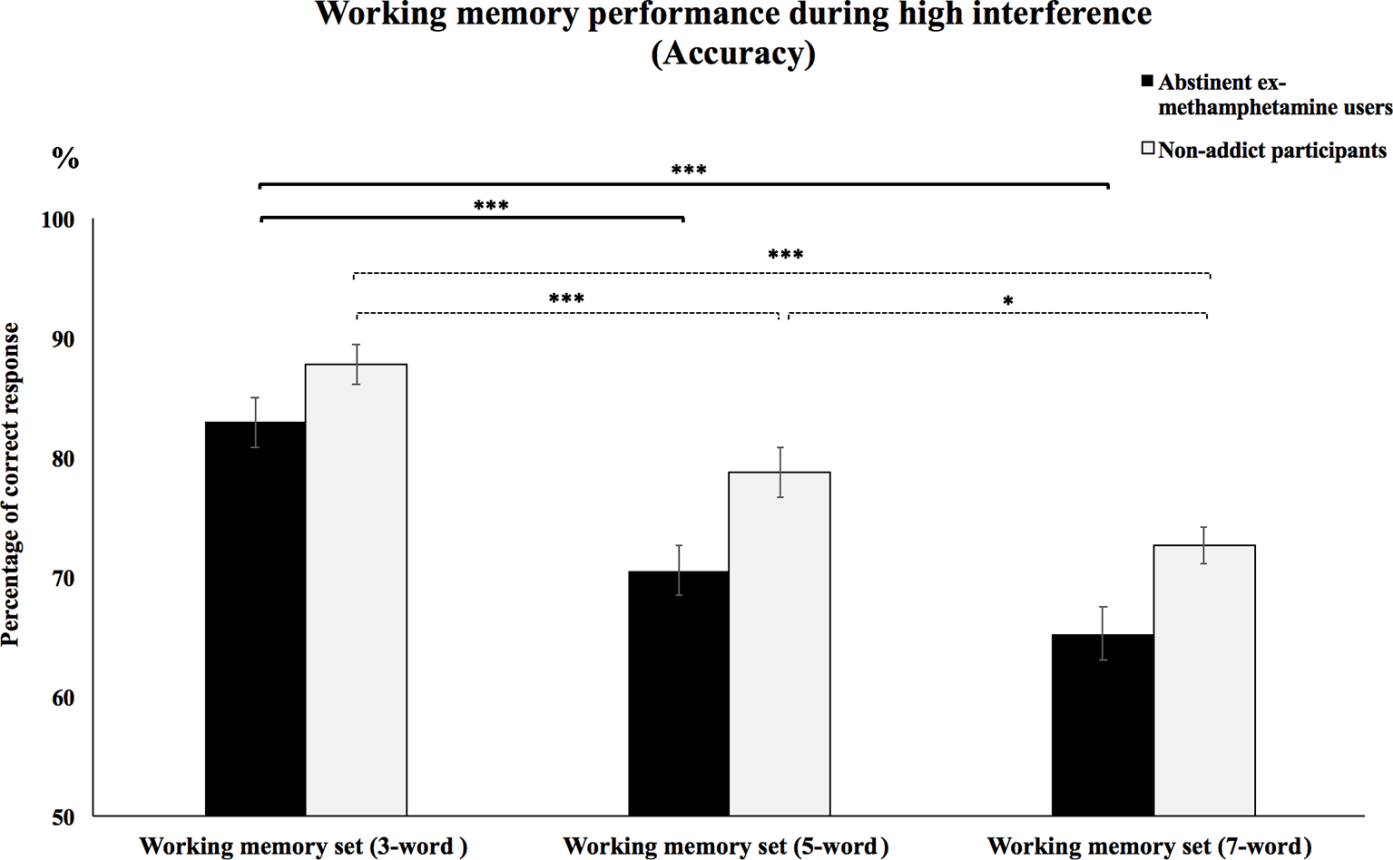
Working memory performance during high interference (accuracy).Mean accuracy of abstinent ex-methamphetamine users and nonaddict control group in recognition of words from the 3-word working memory sets was compared to the five- and seven-word working memory sets during trials with high interference. Error bars indicate standard error of the mean ***p ≤ 0.001 and *p ≤ 0.05.

Repeated-measures ANOVA test showed no significant interaction effects between WM load and subject group on the accuracy of high interference condition (*F*(2, 116) = 2.91, *p* = 0.058). However, priori hypotheses were tested with planned contrasts, and the type I error rate was controlled for using the Bonferroni correction for multiple comparisons. Planned contrasts analysis revealed that the mean accuracy of the methamphetamine user group significantly increased during the performance of the 3-word WM set compared to performing the 5- and 7-word WM sets (*p*’s < 0.001). However, no significant difference was observed in mean accuracy during the 5-word WM set compared to mean accuracy when performing the 7-word WM sets (*p* > 0.2). Mean accuracy of the control group during performance of the 3-word WM set compared to the 5- and 7-word memory sets was significantly increased (*p*’s < 0.001). Besides, mean accuracy was significantly increased when comparing the 5-word WM sets to the 7-word sets (*p* = 0.03) (**Figure 4**).

Demographic information is summarized in **Table 1**.

## Discussion

The novel finding of the current study is that abstinent ex-methamphetamine users compared to a nonaddict group showed a bias toward methamphetamine-related stimuli only in in low WM load conditions (3-word WM sets). These results suggest that increasing the load of WM might reduce the effect of interference. In addition, there was no statistically significant difference in WM performance between all three WM load conditions during trials with high interference between both groups. These findings suggest that increasing the load of WM shields the effect of interference. Besides, attentional bias toward methamphetamine-related stimuli, which were presented during the encoding phase of WM, may contribute to optimal WM performance and may increase the availability of the shared cognitive resources.

### Bias Caused by Difference Sources

#### The First Bias

The results showed that abstinent ex-methamphetamine users showed a bias (i.e., the summation of WM interference bias and WM bias) during low WM load (three words) task performance but not during high load WM (five and seven words) compared to the nonaddict group. The impulsive process may trigger cognitive biases such as attentional bias for drug-related stimuli (14, 15, 18). Studies showed that the use of drugs develops a specific reward system in the brain by releasing dopamine in mesolimbic brain areas, which in turn enhance learning by conditioning (26, 50–52). Attentional bias toward drug-related stimuli results in prolonging the disengagement of attention from those stimuli, leading to increased RTs (20, 51, 53). However, following the views that WM protects bias toward distractors, we expected to find a modulation over distraction (i.e. methamphetamine words) under higher WM loads (29).

#### The Second and Third Bias

Results from the current study showed no significant differences between groups for the second and third biases (i.e., WM interference bias, WM bias). These results can best be considered with some possible explanations:

- Abstinent ex-methamphetamine users are probably motivated to quit or stay abstain from drugs (54, 55). This motivation leads individuals to develop avoidance strategies to cope with tempting stimuli, which meanwhile might be effortful for them (54, 56). For example, heavy alcohol drinkers showed an attentional bias toward alcohol-related stimuli, while abstaining alcohol-dependent individuals avoided such stimuli (24, 57, 2, 55). Besides, studies have also indicated that presenting drug-related stimuli long enough (e.g., 500 ms) can give patients enough time to use efficient avoidance strategies; therefore, the effect of attentional bias might be reversed and away from drug-related stimuli (24, 57).
- The cognitive effort, which has been closely coupled with concepts of attention, difficulty in concentration, and motivation, can also explain current results (58). Cognitive effort can modulate the cognitive resources dedicated to a particular task (59). Indeed, to accomplish more demanding tasks, we have to exert more effort, which leads to a reduced effect of distractors (59, 60). Although we did not measure cognitive effort during the task, abstinent ex-methamphetamine users might invest more effort to perform WM task.
- Drug-related stimuli might elicit an attentional bias toward those stimuli, resulting in an interruption in the performance during the ongoing task. However, it is also possible that drug-related stimuli can elicit a motor response to provide fast and necessary reactions, resulting in avoiding the effect of distractions (61, 62). Besides, according to the literature on anxiety, the shorter RTs for threat stimuli in threat-neutral pairs could indicate an attentional bias away from the threat (61–63). Therefore, it is postulated that salient methamphetamine-related stimuli might lead to increased anxiety, resulting in quicker responses.
- The reflective process can moderate the impact of the impulsive process by emphasizing the effect of WM capacity (top-down process) (14, 17, 18, 64–67). For example, studies have supported the moderating effect of WM capacity on alcohol abuse (27, 64). These results indicated that individuals with a lower WM capacity show strong correlations between implicit alcohol associations and the use of alcohol (14, 27, 64). Although traditional models of impulse control have emphasized the adverse effect of increasing cognitive load on self-regulation, emotion-related studies have supported the idea that increased cognitive load can inhibit feelings of temptation (68–71). Regarding this issue, attention toward an emotional target is automatic (i.e., fast and involuntary), but it is also resource-dependent (71–73). It means that an increased cognitive load may lead to a decrease in the motivation to process task-irrelevant stimuli despite their saliency and associated feelings of temptation (71, 74). For example, categorizing the gender of angry faces compared to happy faces—as an index of selective attention to threatening information—was slower during the mental rehearsal of a one-digit number (low cognitive load) compared to the rehearsal of an eight-digit number (high cognitive load) (74). The bottom line is that there is bias variability in the addiction literature, which makes the basic mechanisms still unclear, but this bias might reflect variations in top-down cognitive control (47, 62, 75). In addition to the emotion-related studies, the WM theory proposed by Andrade et al. (76) also supports the current findings. This theory suggested that retrieving information from WM requires WM capacity, but if the capacity of WM (resource) is occupied during memory reactivation, the emotionality and saliency of new information will be decreased, which will result in updating that information into a less emotional form (67). For example, studies have shown that using a high WM load task (visual-spatial task) during the retrieval of drug-related information could decrease cigarette (77) and food cravings (67, 78).

In summary, studies have indicated that WM and attention processes recruit similar neural networks and share common cognitive resources (79–81). In our case, attentional bias to the methamphetamine-related stimuli, particularly during high WM loads, may bring gain in WM performance (particularly during the encoding phase) and may increase the availability of cognitive resources. The result of the first bias during a task with low WM load indicated that attention was directed toward salient methamphetamine-related stimuli. On the other hand, performing tasks with high WM loads inhibited the effect of bias in abstinent ex-methamphetamine users. Regarding the results of the second and third bias considered, we suggest that presenting methamphetamine-related stimuli and increasing loads of WM were helpful for the experimental group to inhibit the effect of interference. In line with the dual-process models of addiction, which is focused on the trade-off between impulsive and reflective processes, these findings suggest that WM engagement and increased WM load improved avoidance strategy, possibly through reflective processes (71).

### The Effect of Different WM Loads on the Performance of Participants During High Interference Trials

Our findings showed that increasing WM load resulted in increased RTs and decreased accuracy in both the abstinent ex-methamphetamine and nonaddict groups. These results were supported by previous studies showing that increasing WM load could decrease WM performance (82–85). However, there was no significant difference between groups, which contradicted our hypothesis.

There are contradictory findings regarding the effect of methamphetamine on WM performance. On the one hand, some studies have revealed overall cognitive deficits in the domains of verbal memory, WM, executive function, and social cognition in methamphetamine users (9, 10, 20, 86–89). For example, one study indicated that chronic methamphetamine users showed a deficit in some CogState battery domains (i.e., evaluated seven cognitive domains including WM) and poor psychological well-being (88). Studies have also revealed that methamphetamine users had deficits in brain function in areas including the dorsolateral prefrontal cortex during performing cognitive tasks that assess executive function (WM) (20, 87). Activity in this brain area can support WM performance and allocation of attentional resources (36, 90, 91).

However, in line with our results, some studies have shown no significant difference in WM performance of abstinent ex-methamphetamine compared to the nonaddict group (30). For example, Boileau et al. (92) asked methamphetamine users and subjects from a control group to perform different cognitive tasks including WM, attention/psychomotor function, and immediate and delayed memory tasks. Their results showed that there was no significant difference in WM performance between both groups, but that in attention/psychomotor function and delayed memory tasks, methamphetamine users showed a deficit. Another study employed attention/psychomotor function tasks (e.g., Stroop), learning/memory tasks, WM tasks, response inhibition tasks, and set-shifting/executive function tasks for both methamphetamine users and a control group (93). The findings indicated no significant difference between groups for all cognitive tasks. Similar results were observed in other studies as well (94, 95); for a review of this topic, see Hart et al. (30).

The use of different kinds of WM tasks in different experiments might explain these contradictory findings. In our study, we utilized the modified Sternberg task, in which methamphetamine-related stimuli were presented during the encoding phase of WM. These salient methamphetamine-related stimuli might cause attentional bias leading to attentional capture and eventually contributing to better performance despite having an interference bias. Indeed, attentional bias to methamphetamine-related stimuli might highlight those stimuli in WM, resulting in enhanced WM performance. Also, type of distractors might be an important factor to explained contradictory findings. For example, the amount of physical separation between targets and distractors might modulate the effects of load on distraction (29).

The availability of cognitive resources for optimal task performance and inhibiting the effect of interference is critical, particularly when WM is highly loaded or saturated (29). Our findings suggest that cognitive resources might be available as they are not dominated by task demands, resulting in optimal performance. Besides, bias toward the methamphetamine-related words (which were task-relevant information) might facilitate the WM process.

### Limitations and Future Directions

Only male participants were recruited in the present study to minimize the effect of potentially confounding factors. One noteworthy and currently unexplored direction for future studies might be to examine gender differences. Moreover, participants with the mean of nearly 17-day abstinence from methamphetamine use were recruited. We chose this sample to assess the effect of short time abstinence from methamphetamine on WM performance. However, it is still unclear what may be the effect of long-term abstinence from methamphetamine use on cognitive function. For example, one study revealed that enhanced performance on tests of verbal memory and executive function was observed after approximately 6 months of abstinence from methamphetamine use. In line with this idea, some studies have examined the role of duration of abstinence from methamphetamine use on cognitive function (96, 97). They showed that prolonged greater duration of abstinence from methamphetamine use resulted in better cognitive performance (96–98). Future studies might consider the effect of long-term abstinence from methamphetamine use on WM biases and WM capacity. In addition, this study did not include a sample of active methamphetamine users who do not want to quit drug use, due to the difficulty in performing the modified Sternberg task. Future studies might add this group to compare the effect of motivation to quit on WM performance between abstinent and active groups. Due to the size of the center and the limited-time permission we have for our study, we could not recruit more participants. In several tests, we realized that the power of analysis is below the optimal level, and for some interactions, there was only a trend toward significance. In the future studies hiring complex tasks, more participants should be recruited to have enough power to run all the required analyses properly. This study sought to examine the neurobiological substrates of the interaction between WM bias, WM capacity, and interference effect using a complex span task in methamphetamine users.

## Conclusion

We investigated the impulsive effects of methamphetamine-related stimuli on WM performance in abstinent ex-methamphetamine users. The experimental group demonstrated bias toward methamphetamine-related stimuli during a task with low WM load (three words) but not while performing tasks with higher WM loads (five and seven words). This result suggests that increasing WM load may provide an efficient buffer against attentional capture by salient stimuli (i.e., methamphetamine-related words). In line with this findings, investigating the effect of increasing WM load on the performance of abstinent ex-methamphetamine users (i.e., WM capacity) showed that increasing WM load had no significant effect on WM performance of abstinent ex-methamphetamine users compared with the control group. These findings suggest that increasing WM loads modified the impact of the interference bias. Besides, presenting methamphetamine-related stimuli facilitated their encoding due to bias toward task-relevant stimuli.

This finding has an important implication, suggesting that performing concurrent demanding tasks may reduce the power of salient stimuli and thus improve the efficiency of emotion regulation strategies. Further investigation on the interactions between WM interference bias, WM bias, and WM capacity may lead to the development of better tools and alternative therapies, including WM training, for the treatment of addiction.

## Ethics Statement

All experimental procedures corresponded to the standards set by the latest revision of the Declaration of Helsinki and were approved by the ethical committee of the Institute for Cognitive Sciences Studies, Tehran, Iran. All participants provided written informed consent, acknowledging their right to withdraw from the experiment without prejudice.

## Author Contributions

ZD contributed to all aspects of the research. HE contributed to experimental design and data interpretation. HP contributed to experimental design and data interpretation. AK contributed to all aspects of the research.

## Conflict of Interest

The authors declare that the research was conducted in the absence of any commercial or financial relationships that could be construed as a potential conflict of interest.

## Abbreviations

WM, Working Memory; RTs, Reaction Times.
